# Supplementary material for: A microfluidic platform integrating functional vascularized organoids-on-chip
Source: Nat Commun. 2024 Feb 16;15:1452. doi: 10.1038/s41467-024-45710-4 (PMC10873332; doi:10.1038/s41467-024-45710-4)
Supplement: Supplementary file 1 — Supplementary Information [file 41467_2024_45710_MOESM1_ESM.pdf]

SUPPLEMENTARY INFORMATION

A microfluidic platform integrating functional vascularized organoids-on-chip

Authors

Clément Quintard[1,2,3], Emily Tubbs[1], Gustav Jonsson[4,5,6], Jie Jiao[3], Jun Wang[3], Nicolas Werschler[7], Camille Laporte[1,2], Amandine Pitaval[1], Thierno-Sidy Bah[8], Gideon Pomeranz[9], Caroline Bissardon[2], Joris Kaal[2], Alexandra Leopoldi[4,6], David A. Long[9], Pierre Blandin[2], Jean-Luc Achard[10], Christophe Battail[8], Astrid Hagelkruys[4,6], Fabrice Navarro[2], Yves Fouillet[2], Josef M. Penninger[3,4,6,11*], Xavier Gidrol[1*]

Affiliations

[1] Univ. Grenoble Alpes, CEA, IRIG/BGE, BIOMICS, 38000 Grenoble, France

[2] Univ. Grenoble Alpes, CEA, LETI, DTBS, 38000 Grenoble, France

[3] Department of Medical Genetics, Life Sciences Institute, University of British Columbia, Canada

[4] Institute of Molecular Biotechnology of the Austrian Academy of Sciences, IMBA, Dr. Bohr-Gasse 3, 1030 Vienna, Austria

[5] Vienna BioCenter PhD Program, Doctoral School of the University of Vienna and Medical University of Vienna, 1030, Vienna, Austria

[6] Eric Kandel Institute, Department of Laboratory Medicine, Medical University of Vienna, Vienna, Austria

[7] School of Biomedical Engineering, University of British Columbia, Vancouver, BC, Canada

[8] Univ. Grenoble Alpes, CEA, IRIG, BGE, Gen&Chem 38000 Grenoble, France

[9] Developmental Biology and Cancer Programme, UCL Great Ormond Street Institute of Child Health, London, UK

[10] Université Grenoble Alpes, CNRS, Grenoble INP, LEGI, 38000 Grenoble, France

[11] Helmholtz Centre for Infection Research, Braunschweig, Germany

Corresponding authors

Josef M. Penninger, Xavier Gidrol

*E-mail: josef.penninger@ubc.ca; xavier.gidrol@cea.fr

Supplementary Figures 1-14

Supplementary Notes 1-3

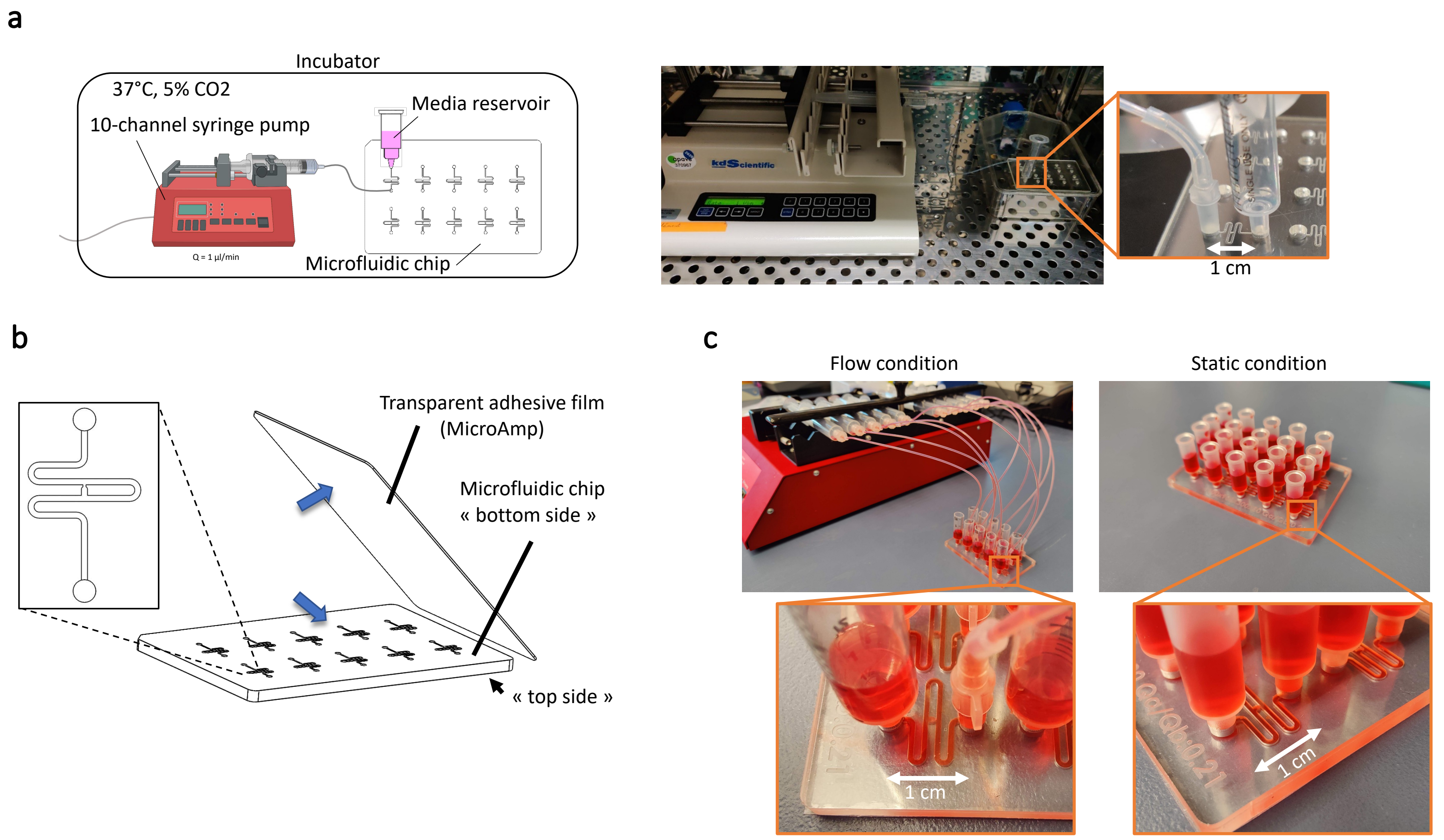

d

Measurements on trap dimensions

	LB [µm]	wI [µm]	wB [µm]	wA [µm]	h [µm]
Average of card 1	82 ± 7	508 ± 6	162 ± 7	402 ± 4	395 ± 15
Average of card 2	83 ± 6	514 ± 6	163 ± 10	408 ± 7	385 ± 24
Average of card 3	78 ± 9	510 ± 4	168 ± 7	405 ± 4	395 ± 11
Average of card 4	78 ± 6	516 ± 4	170 ± 10	407 ± 8	400 ± 15
CAD file	90	500	150	400	400
Average difference between cards and CAD file	-10 ± 3	12 ± 4	16 ± 4	6 ± 3	-6 ± 6

Trap dimension labelling

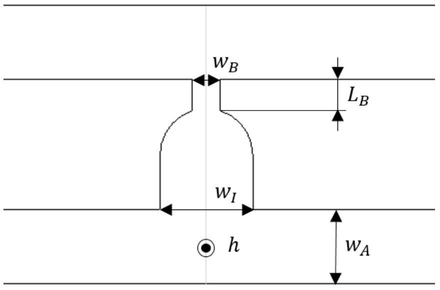

Supplementary Fig. 1. Experimental set-up for the continuous perfusion of the organoids-on-chip. a, Sketch and photograph of the microfluidic platform. Using a 10-channel syringe-pump, the flow can be monitored in the 10 microchannels of the chip. For a better visualization, only one microchannel was connected to the syringe pump on this photograph (diagram partly created with BioRender.com). **b,** Assembly of the microfluidic chip. The microchannels were closed using a transparent MicroAmp adhesive film. **c,** Photographs of the microfluidic platform with 10 microchannels connected in parallel, under flow and static conditions. **d,** Dimensioning of microfluidic chips. The critical dimensions for organoid trapping were measured within each microchannel to guarantee the operational effectiveness of the hydrodynamic trapping aspect. Data represents mean ± s.d. from measurements taken across the 10 microchannels of each microfluidic chip.

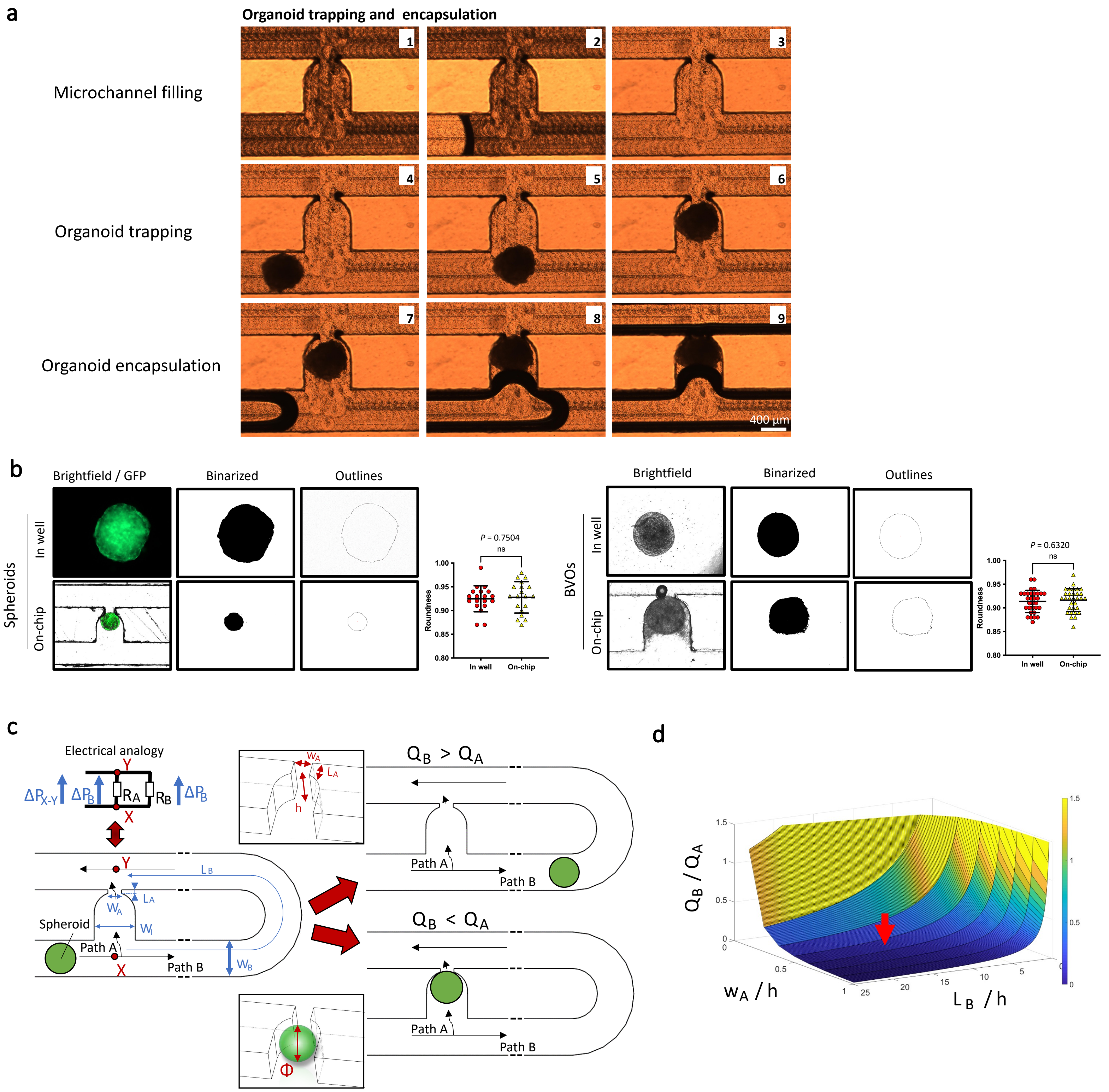

Supplementary Fig. 2. Experimental validation of organoid trapping and encapsulation. a, Stills from a movie of the experimental trapping of one organoid was recorded at 15 fps using an inverted microscope (bright field). First, the hydrogel fills the microchannel (microchannel filling, steps 1-3). Then, the organoid is trapped in the U-cup shaped area (organoid trapping, steps 4-6). Last, the air pushes the hydrogel towards the outlet of the microchannel (organoid encapsulation, steps 7-9). The hydrogel-air interface can be seen in black due to the difference in the refractive index. **b,** Evaluation of the roundness of spheroids (n = 19) and organoids (n = 31) before and after on-chip encapsulation. The spheroids and organoids were selected according to their spherical shape (roundness above 0.8. Spheroids (n = 19): roundness 0.92 ± 0.03. Organoids (n = 31): roundness 0.92 ± 0.02). They were precisely positioned in the trap site without any apparent morphological alteration, with a roundness maintained above 0.8 (Spheroids (n = 19): roundness 0.93 ± 0.03. Organoids (n = 31): roundness 0.92 ± 0.02). Data represent mean ± s.d. **c,** A comprehensive perspective of the trapping unit, highlighting the conditions necessary for successful organoid trapping. **d,** Surface Q_B/Q_A as a function of L_A/h and w_A/h (in the case of a circuit dimensioned with $w_A = h = 400$ µm). The red arrow indicates the geometrical parameters chosen in practice, $L_A/h = 0.25$, $w_A/h = 0.5$ resulting in $Q_B/Q_A = 0.15$ (created with Matlab). Source data are provided as a Source Data file.

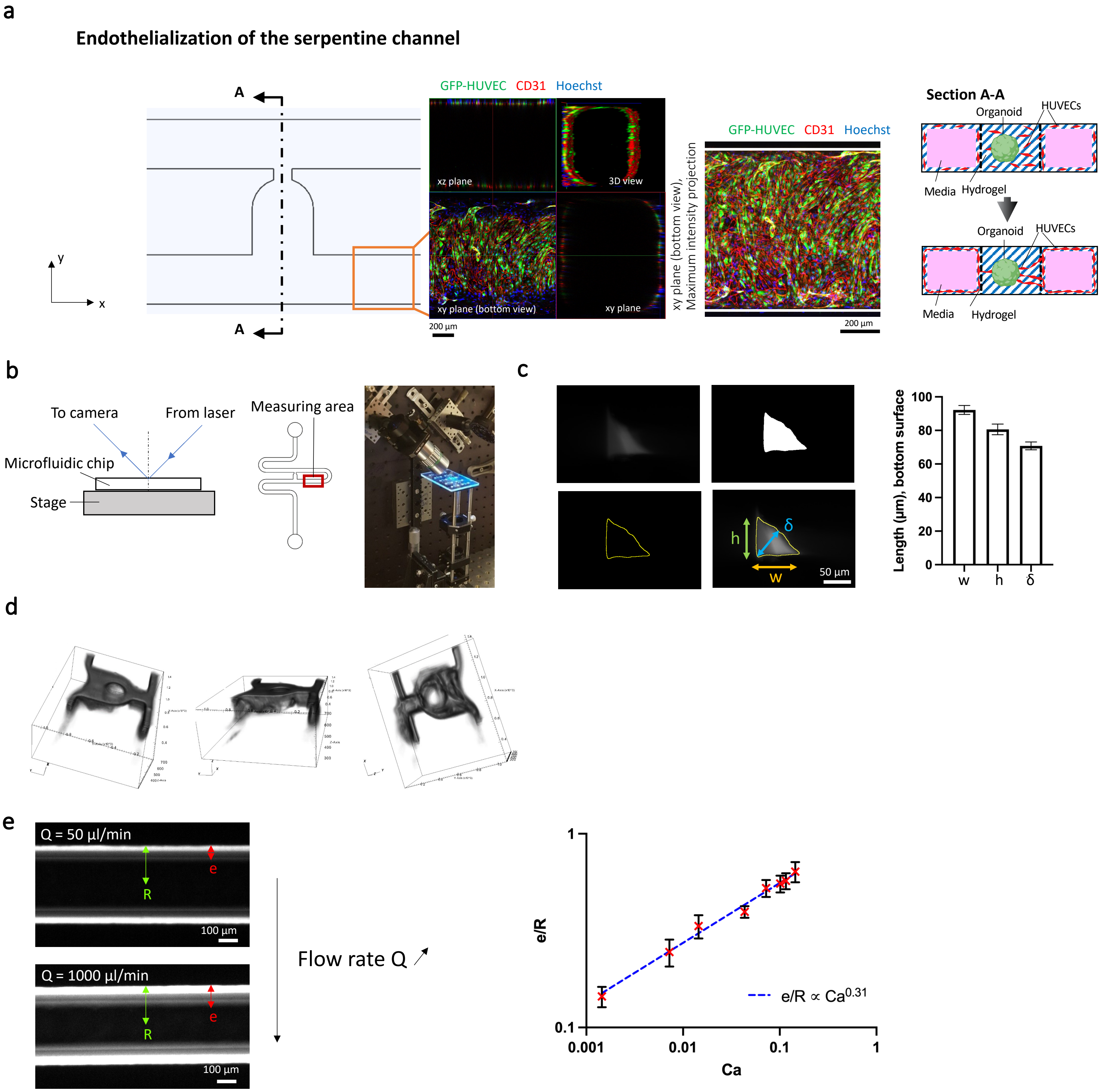

Supplementary Fig. 3. Gel loading in the microfluidic chip using the Landau-Levich-Bretherton coating. a, Schematic diagram and experimental results of the complete endothelialization of the main serpentine microchannel taking advantage of the Landau-Levich-Bretherton phenomenon. **b,** Sketch and photograph of the optical set-up for light sheet imaging of the chip. A stack of 100 images was acquired in the measuring area to quantify fluorescent hydrogel deposition with a step size of 10 microns along the microfluidic channel using an automated stage. **c,** Images of the hydrogel deposition within one microchannel corner showing raw image, Otsu-based segmentation mask image, segmentation contouring and superposition of raw image and mask. The dimensions of the remaining hydrogel corners in a cross-section of the channel, namely the height (h), the width (w), and the diagonal, Feret's diameter (δ), were collected. The dimensions represent mean ± s.d. and correspond to an average made over 20 images of the bottom surface, acquired at diverse locations along the microfluidic channel. **d,** Representative 3D views of the fluorescent gel distribution within the microfluidic U-cup trap. The lighter part in the center of the trap corresponds to an air bubble trapped within the gel. A python script was used to re-slice the stack of images collected at 45° to a file containing a 3D intensity matrix. The 3D representations of the trap volume were realized using VisIt software. Exposure time: 40 ms. laser power: 3 mW. **e,** Thickness e of the fibrin hydrogel layer following the air bubble passage at flow rate Q, relative to the capillary number Ca, in a straight section of the microchannel of width $2R$ (n = 3 independent microchannels). The gel was visualized using Dextran FITC incorporated into the mixture (top view of the microchannel). Source data are provided as a Source Data file.

a

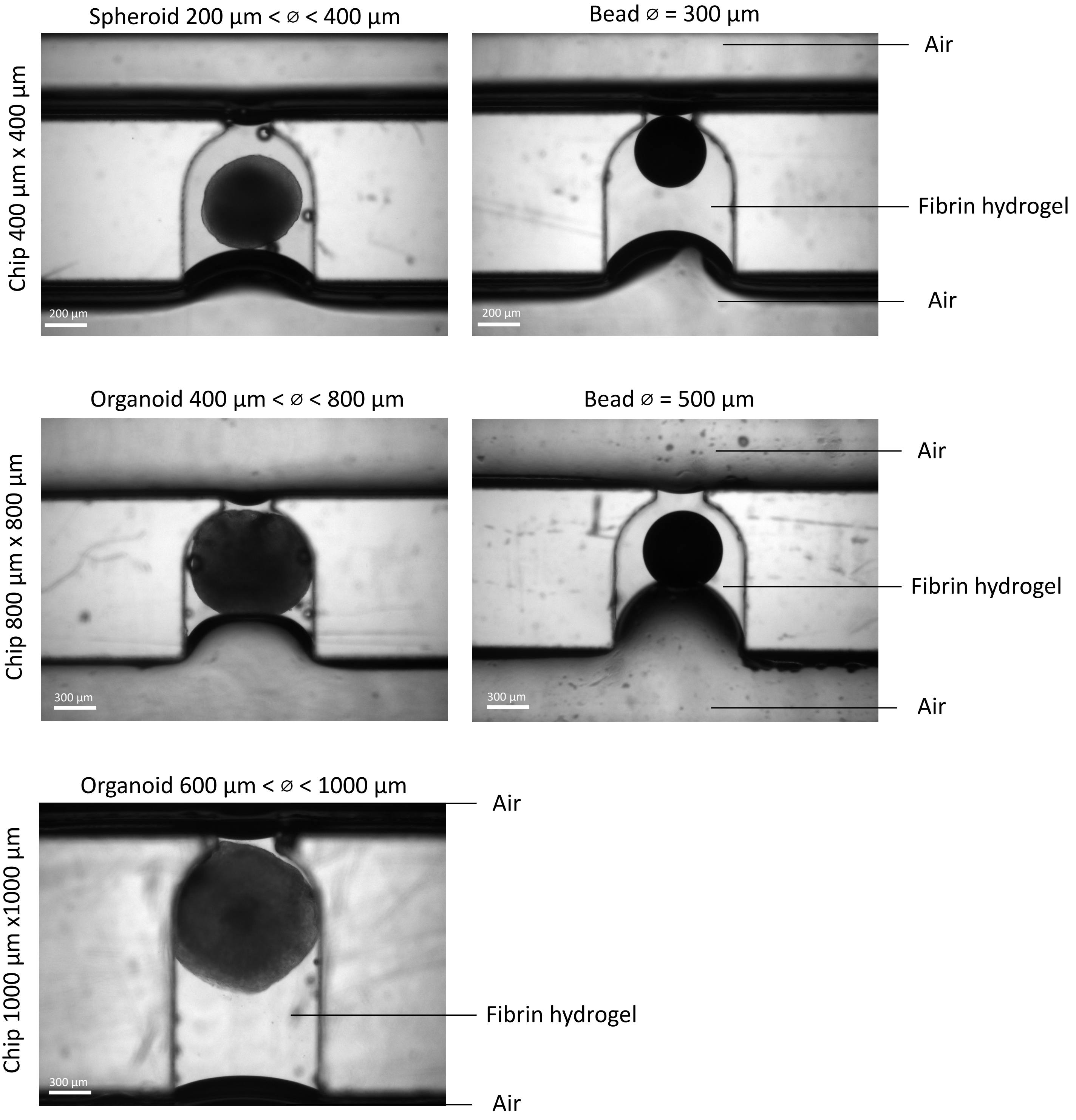

b

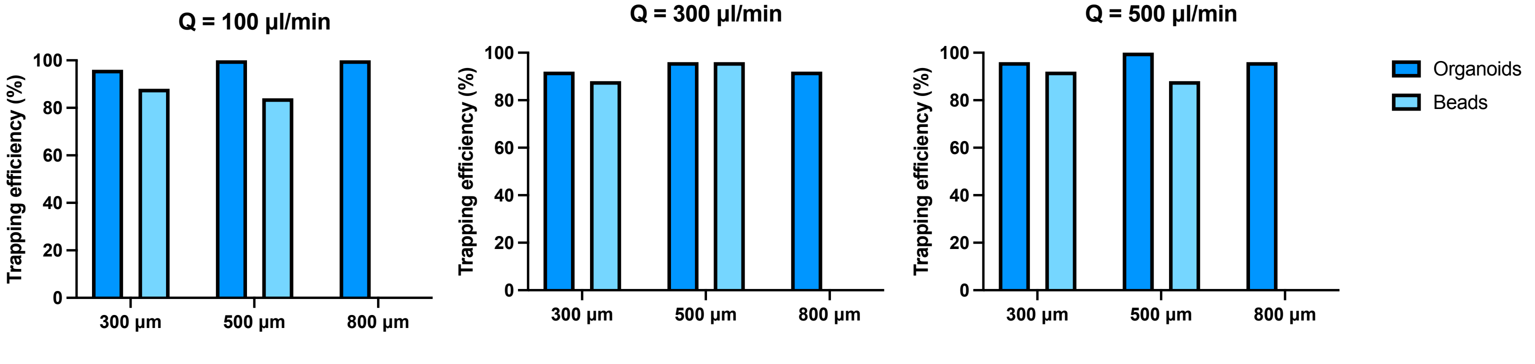

Supplementary Fig. 4. Organoid trapping and encapsulation efficiency. a, Exemplary images show organoids and polymer beads (ChromoSphere™) encapsulated in a fibrin hydrogel, utilizing our method across three distinct chip designs. **b,** Trapping yields at flow rates of 100, 300, and 500 µl/min highlight consistent high trapping efficiency regardless of the flow rate. Trapping efficiency was assessed as number of successfully trapped organoids (or beads) per number of organoids (or beads) loaded on-chip (n = 25 organoids (or beads) per condition).

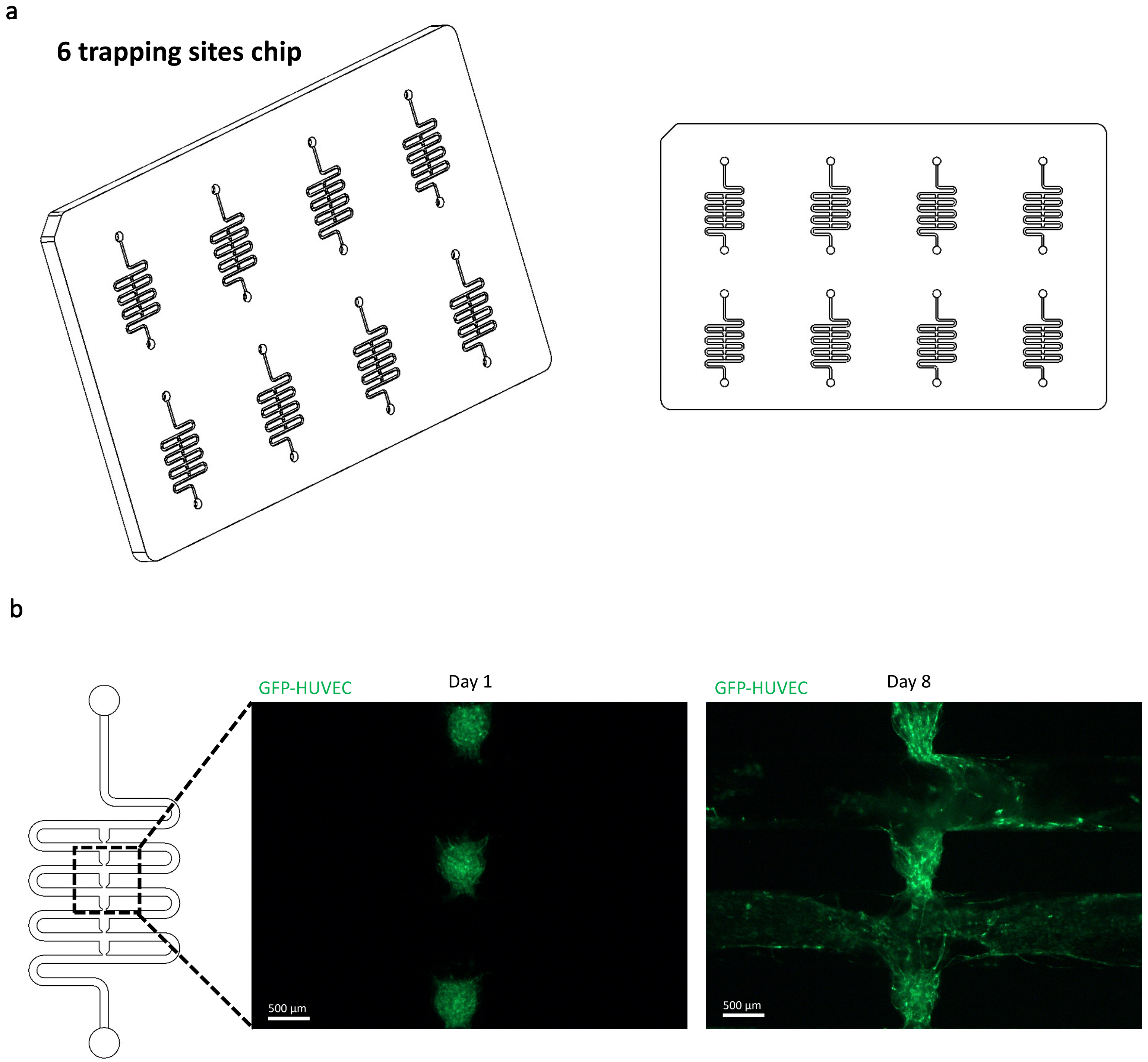

Supplementary Fig. 5. Variation of the original microfluidic design to enable the sequential arrangement of the trapping unit. a, Computer-Aided Design (Solidworks) views showing an alternative design for culturing 6 organoids per microchannel in series. **b,** Experimental results of the mesenchymal spheroids culture using the multiple trapping sites microfluidic chip. Under flow conditions, the spheroids were able to differentiate into vessel like structures and expand in the microchannels.

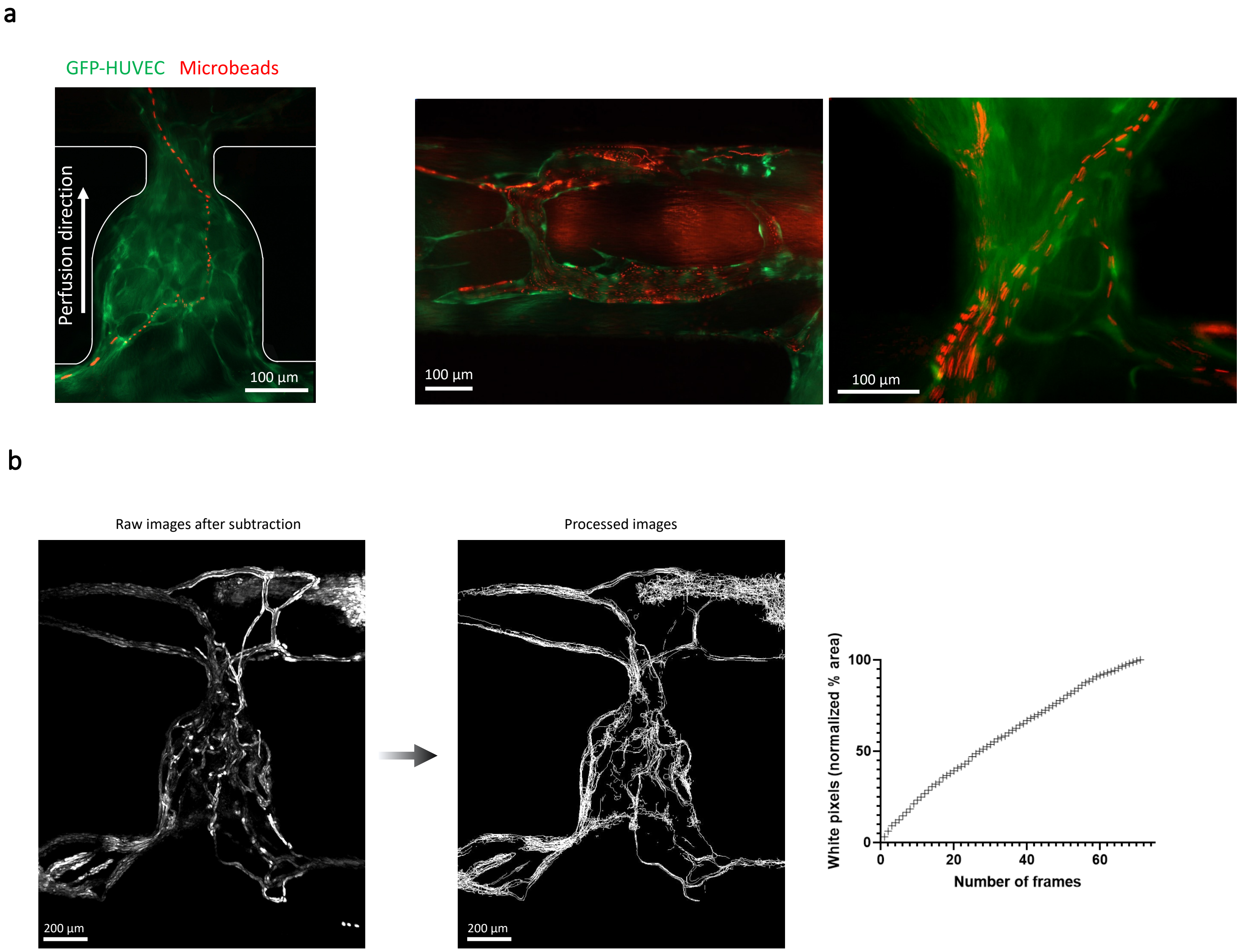

Supplementary Fig. 6. Perfusion of the trapping unit using mesenchymal spheroids. a, Maximum intensity projections over image stacks showing tracking of individual microbeads (red) passing through the endothelial networks (green). For better visualization, the RFP-HUVEC cells are not shown in these images. See Supplementary Video 3 for raw movie. Beads were 1 µm in diameter. **b,** Movies of fluorescent microbeads flowing through mesenchymal vascular network were taken at 15 fps using an inverted microscope. Only the traces of the microbeads have been kept using a subtraction script on ImageJ. The images were then process using the Skeletonize and Binarize features in ImageJ, and superimposed frame by frame. The number of white pixels, corresponding to microbeads movements, steadily increased during the experiment, demonstrating that the microbeads do not prioritize any particular vessel of the microvascular network.

Quantification (Angiogenesis Analyzer plugin)

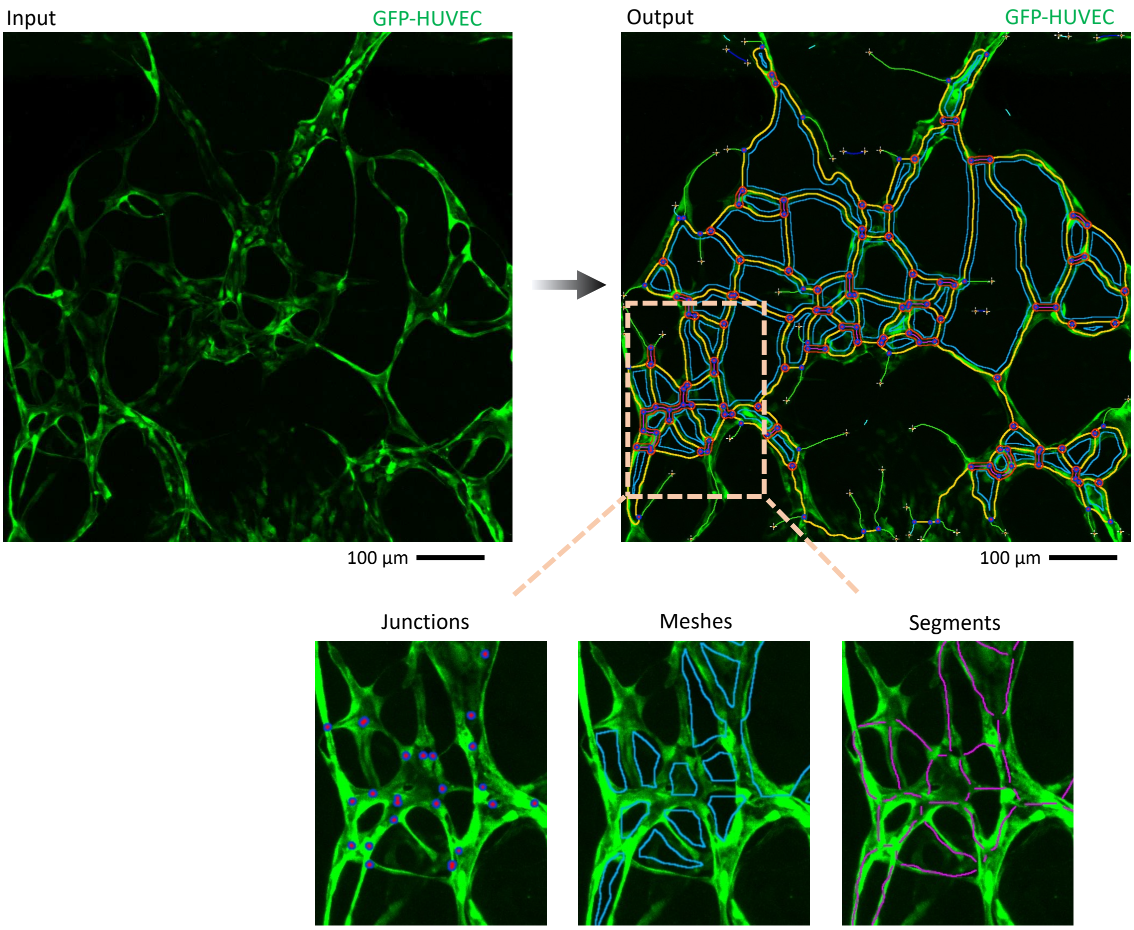

Image name	Microchannel 1
Analyzed area (pixels^2)	1048576
Nb extremities	46
Nb nodes	376
Nb junctions	111
Nb master junctions	54
Nb master segments	100
Tot. master segments length (pixels)	9617
Nb meshes	43
Tot. Meshes area (pixels^2)	327349
Nb pieces	188
Nb segments	147
Nb branches	36
Nb isolated segments	5
Tot. Length (pixels)	12536
Tot. branching length (pixels)	12297
Tot. segments length (pixels)	9430
Tot. Branches length (pixels)	2867
Tot. Isol. branches length (pixels)	239
Branching interval (pixels)	261.944
Mesh index (pixels)	178.093
Mean mesh size (pixels^2)	7612.8

Supplementary Fig. 7. Quantification of endothelial network parameters using the Angiogenesis Analyzer plugin. Confocal z-stacks of the microchannels were taken in the various culture conditions. The z-stacks were taken at the limit of the confocal depth within each sample, nearly 300 µm per sample, which represents approximately the same volume per endothelial network analyzed. Those z-stacks were then flattened in ImageJ to a 2D maximum-intensity projection (as required by Angiogenesis Analyzer). The default settings of the Angiogenesis Analyzer plugin were used. Five different parameters (red dots) representative of the network morphology were used for comparisons in this study.

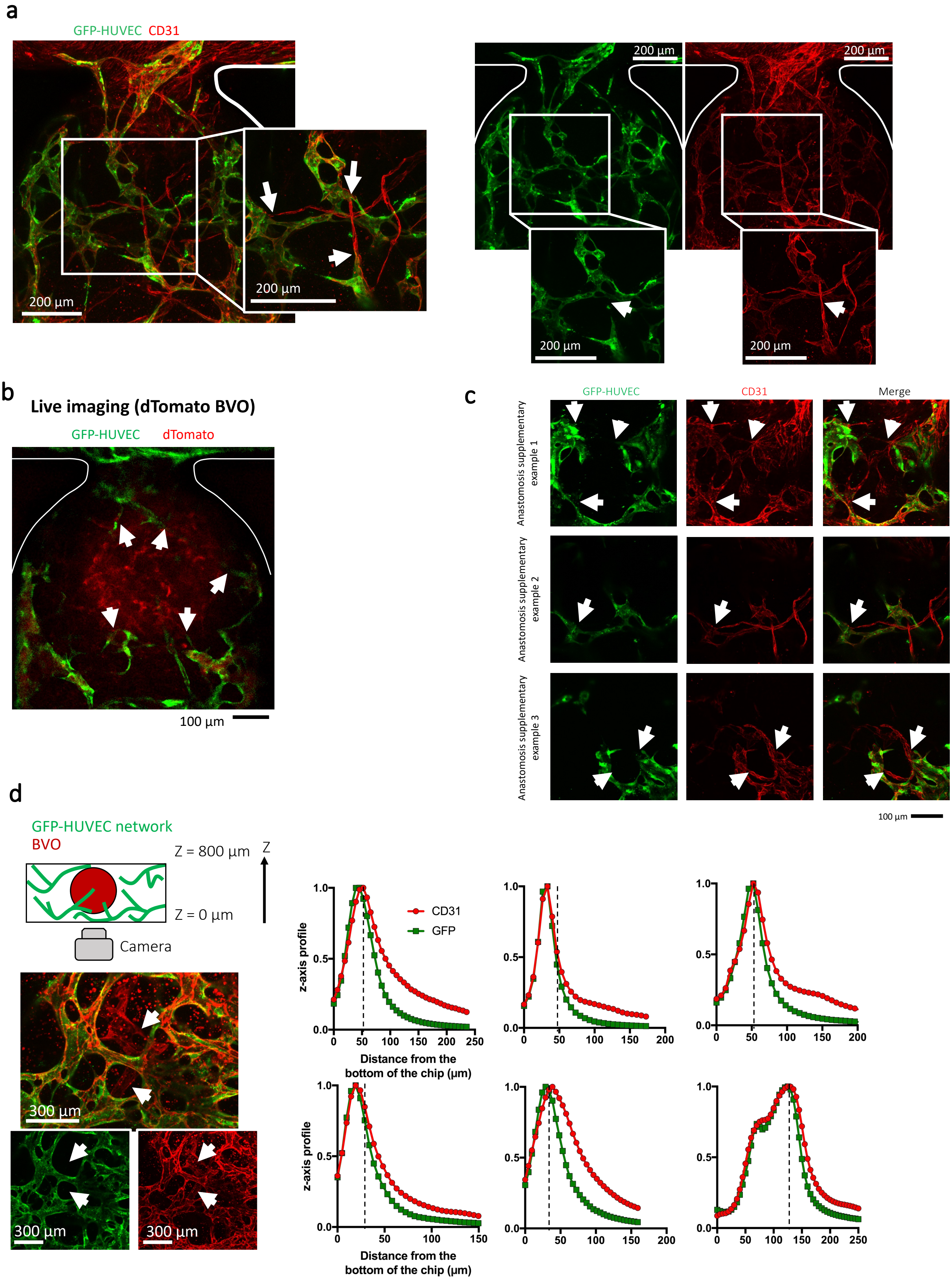

Supplementary Fig. 8. Anastomosis between GFP-HUVEC endothelial networks and blood vessel organoids. a, Confocal z-stacks maximum intensity projection after staining of the microchannels for CD31 expression (red). The BVO vasculature corresponds to $CD31^{+}GFP^{-}$ vessels. Areas where BVO vessels ($CD31^{+}GFP^{-}$) merge with the endothelial HUVEC network ($CD31^{+}GFP^{+}$) are indicated with white arrows. **b,** To explore whether connections were able to form between the HUVEC (GFP green) endothelial bed and the trapped organoid, we used blood vessel organoids (BVO) in which the endothelial lineage was fluorescently labelled with dTomato (CDH5-dTomato). Arrows point at anastomoses. **c,** Higher magnification confocal images of single z-planes at the organoid/HUVEC interface showing connections between the GFP-HUVEC vessels and the organoid vasculature (white arrows). **d,** Analysis of concealed BVOs staining in dense HUVEC networks via z-axis profiling. Confocal z-stack maximum intensity projection after immunostaining to detect CD31+ (red) endothelial cells and detection of GFP+ (green) HUVECs (left). For each graph, a vertical dotted line highlights the divergence of green and red signals, revealing the BVO immunostaining deep inside the microfluidic chip.

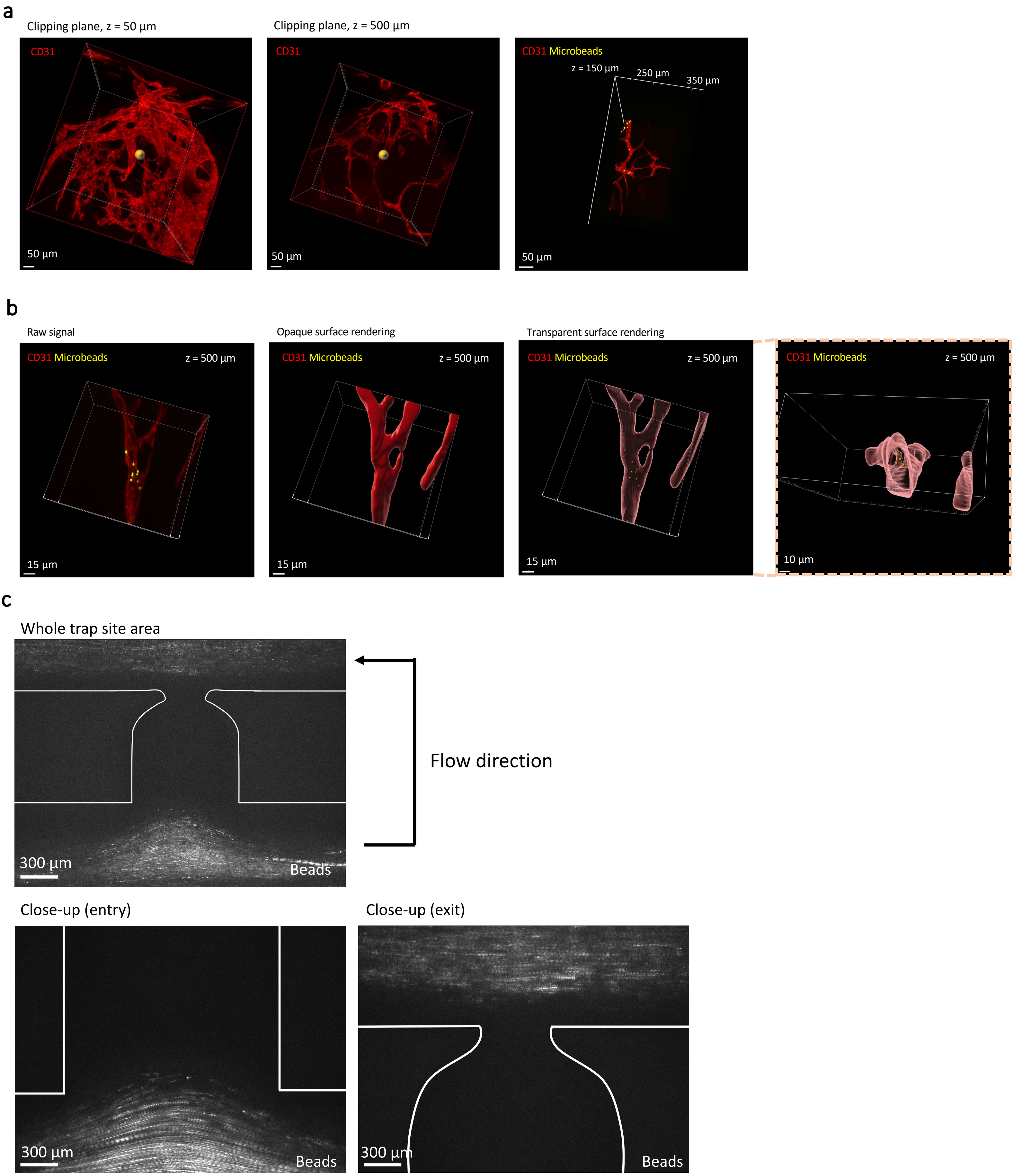

Supplementary Fig. 9. Observation of microbeads stuck inside the inner vasculature of the BVOs after live beads perfusion. a, 3D views from confocal z-stacks of the trapping site highlighting the stained vasculature of a BVO at different z planes (distance from the bottom of the microfluidic chip). **b,** Close-up views from confocal z-stacks on the BVO's vasculature at z = 500 µm from the bottom of the microfluidic chip. Imaris segmentation was used to reveal the presence of fluorescent microbeads located inside the lumen. **c,** Maximum of intensity projection over images stacks highlighting the tracks of the microbeads passing through the loop channel in the absence of HUVEC endothelial network.

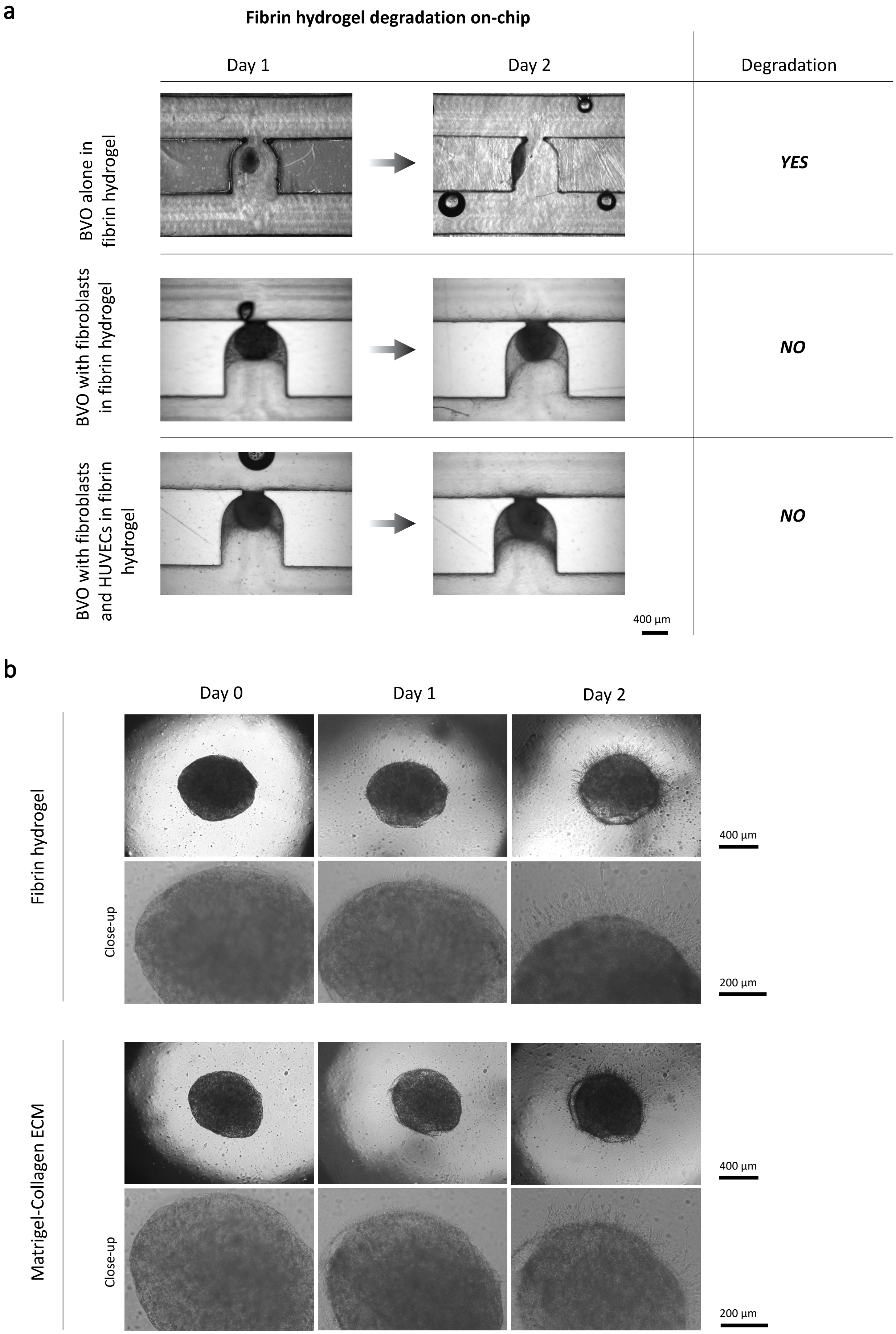

Supplementary Fig. 10. Fibrin hydrogel degradation. a, Representative photographs of the rapid fibrin hydrogel degradation when the BVOs were cultured alone, leading to shrinkage of the organoids. The incorporation of fibroblasts in the hydrogel mix was necessary for maintaining the gel's mechanical durability. **b,** Representative photographs of BVOs cultured alone in fibrin and Matrigel-Collagen I, exhibiting similar growth and sprouting abilities in both matrices.

Tissue extraction of the microfluidic chip

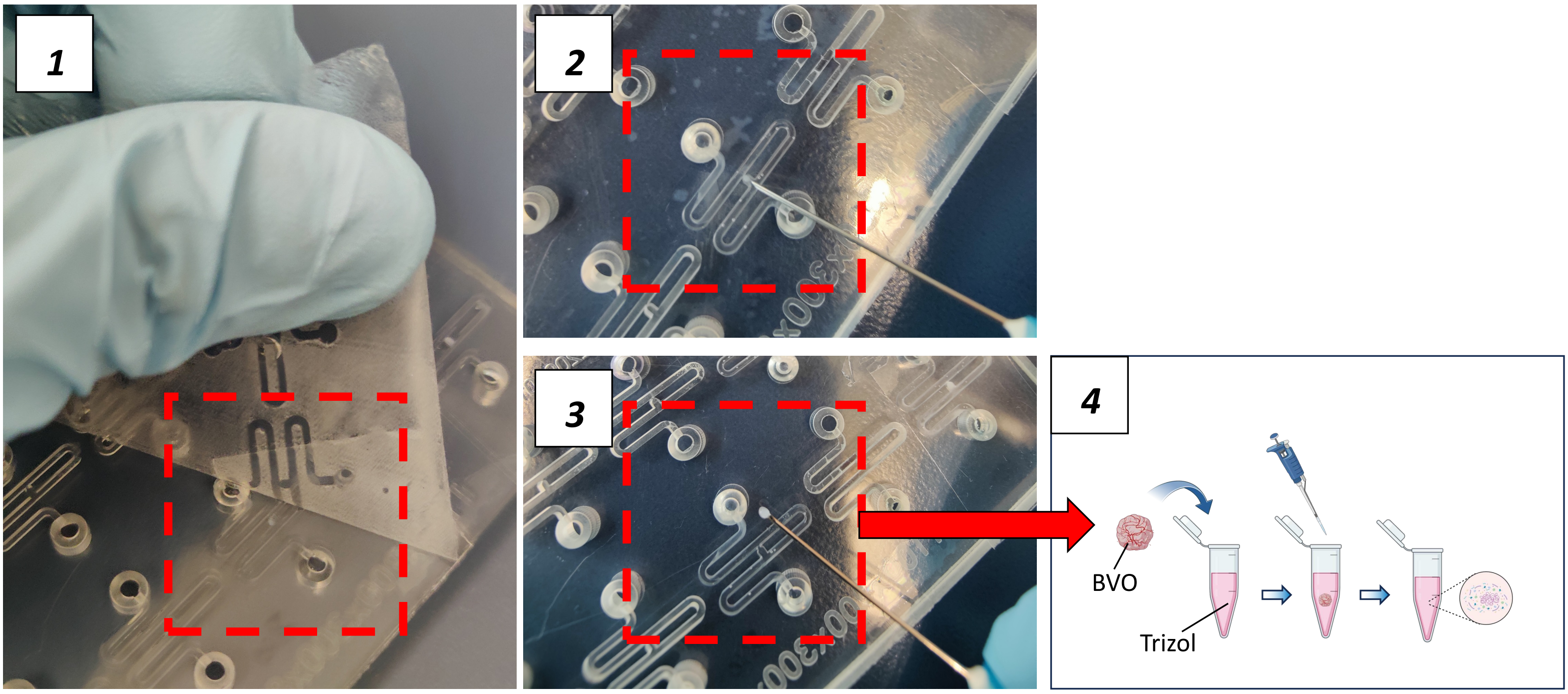

1	Removing of the MicroAmp adhesive film
2	Extraction of the organoid using a needle
3	No surrounding tissue is collected
4	Lysis of the organoid in Trizol

Supplementary Fig. 11. Tissue extraction. Organoid extraction process from the microfluidic device. The microfluidic chip is sealed with a MicroAmp transparent adhesive film, designed for easy removal at any desired timepoint during the experiment. Upon removal of the adhesive film, 3D biological tissues remain positioned within the microchannels. For extraction, a needle is carefully used to pick the organoid without disturbing surrounding tissues, after which it is promptly placed in Trizol for immediate lysis. This design ensures ease and robustness in the organoid retrieval process from the microfluidic platform. Diagram created with BioRender.com

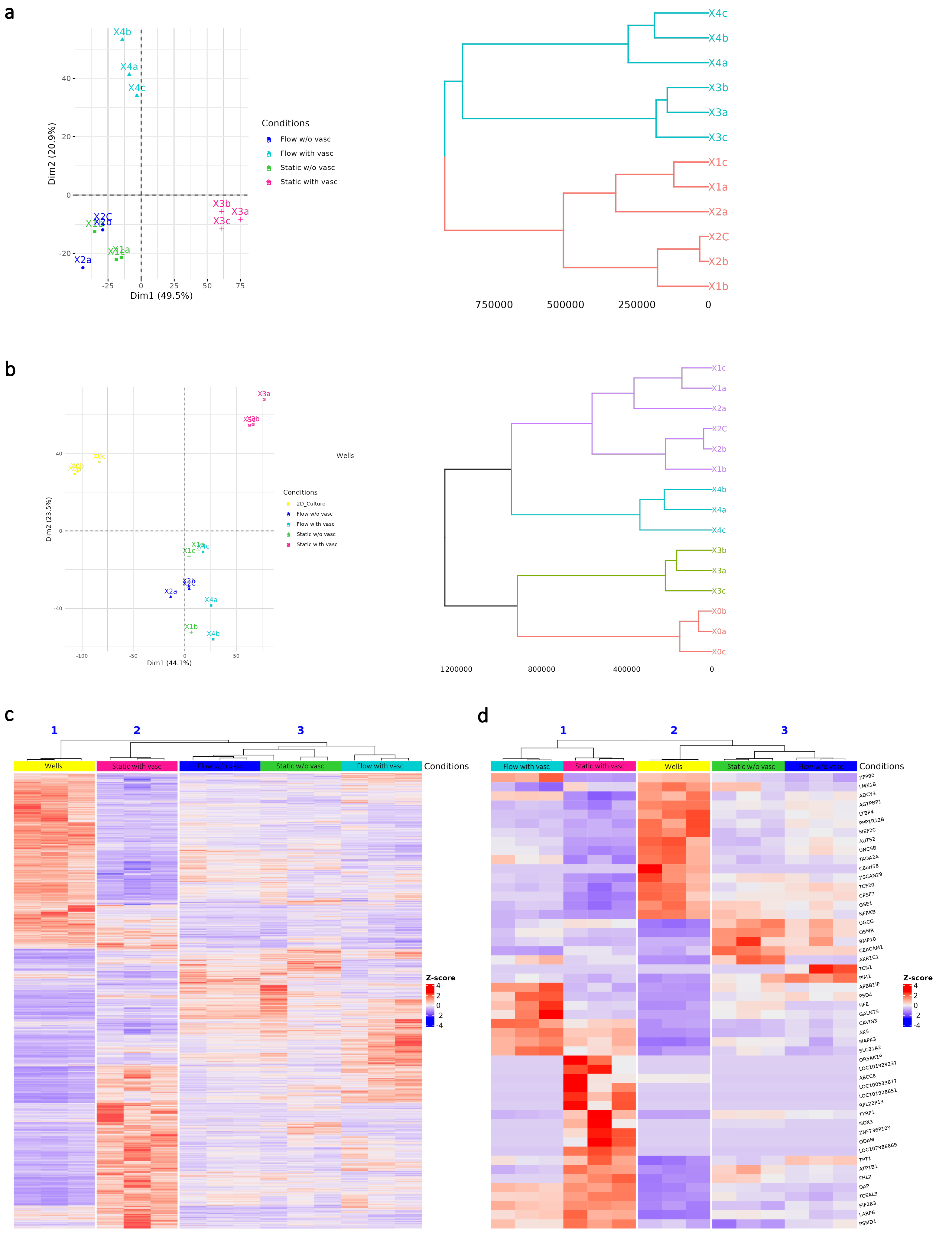

Supplementary Fig. 12. Classification of BVOs transcriptomes. a-b, Hierarchical clustering using Principal Component Analysis (PCA) and dendrogram tools, considering the four chip culture conditions (**a**) and in conjunction with the wells culture conditions (**b**). **c-d,** Semi-supervised classification based on the Kruskal-Wallis test allowing for the visualization of all significantly differentially expressed genes (**c**) and the top 50 most differentially expressed genes (**d**) between all culture conditions used in this study.

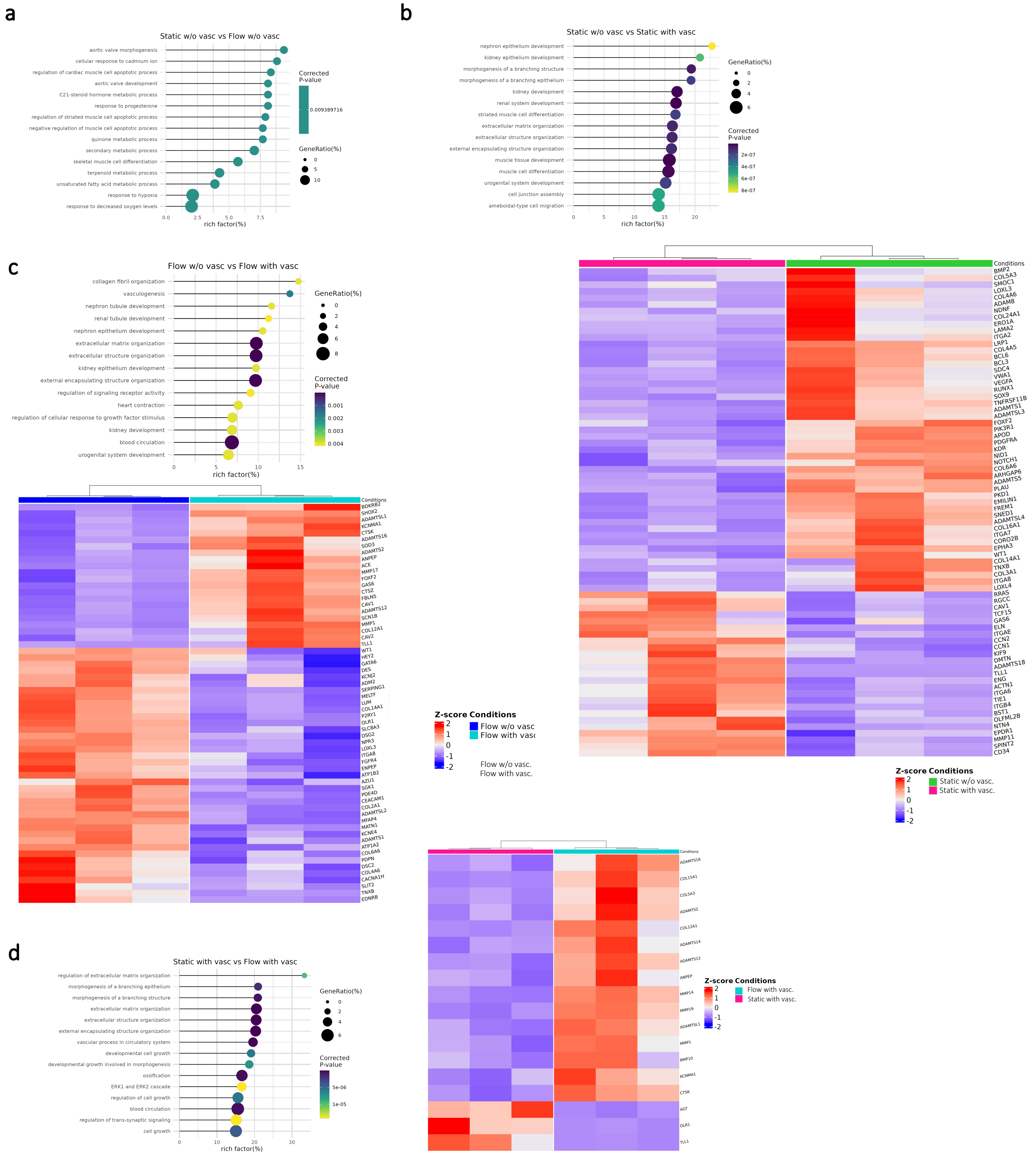

Supplementary Fig. 13. Ontology enrichment analysis of genes differentially expressed between the different conditions of culture on-chip. a-d, Ontology enrichment analyzes were performed using the Gene Ontology - Biological Processes database between the following conditions: “static w/o vasc.” vs “flow w/o vasc.” (**a**), “static w/o vasc.” vs “static with vasc.” (**b**), “flow w/o vasc.” vs “flow with vasc.” (**c**) and “static with vasc.” vs “flow with vasc.” (**d**). Visualization of the top 15 most significantly enriched pathways. The rich factor, on the X axis, corresponds to the ratio between the number of genes differentially expressed for each pathway compared to the total number of annotated genes in the pathway. The gene ratio controls the size of the dots and corresponds to the number of differentially expressed genes for each pathway relative to the total number of differentially expressed genes. Visualization of the top 50 most differentially expressed genes associated with the extracellular matrix organization process between the following conditions: “static w/o vasc.” vs “static with vasc.” (**b**) and “flow w/o vasc.” vs “flow with vasc.” (**c**). Visualization of a gene set involved in extracellular matrix and blood vessel development processes for the conditions “static with vasc.” vs “flow with vasc.” (**d**).

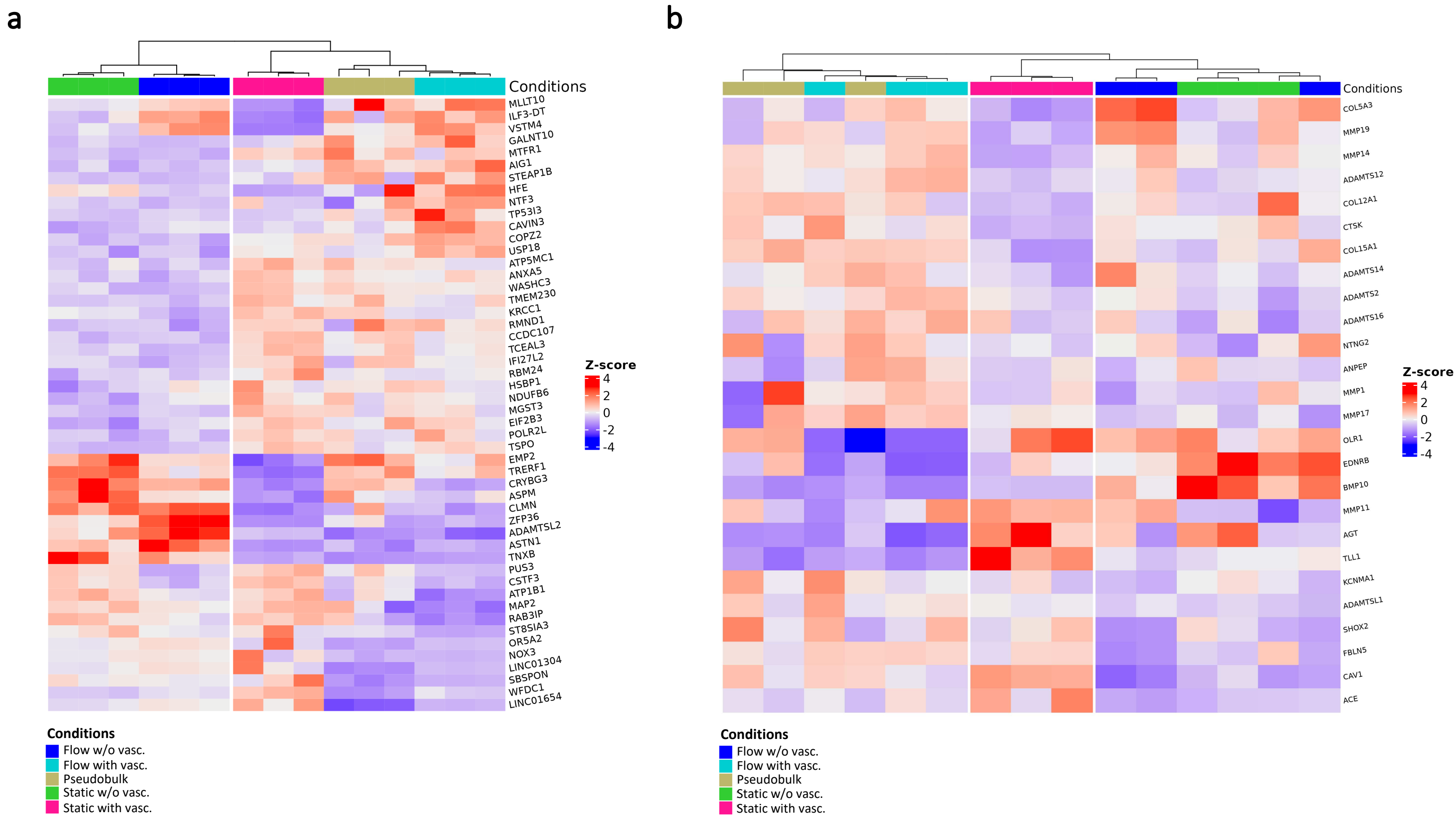

Supplementary Fig. 14. Classification of BVO transcriptomes with a reference tissue simulated from single-cell RNA-seq data of BVOs matured in mice. a, Semi-supervised classification based on the Kruskal-Wallis test of genes significantly differentially expressed across all conditions, followed by visualization of the top 50 most significant genes. **b,** Semi-supervised classification based on the Kruskal-Wallis test of genes significantly differentially expressed across all conditions, followed by visualization of a gene set involved in extracellular matrix and blood vessel development processes.

Supplementary Information

Supplementary Note 1: Organoid trapping and encapsulation (theory supplement)

Upon reaching the trapping unit, the organoid faces two possibilities (refer to Supplementary Fig. 2c). It could either follow path B, bypassing the trap and continuing towards the exit without being trapped, or it could take path A, which leads it to be trapped in the designated area. In order for the hydrodynamic trapping mechanism to be effective, the organoid should predominantly opt for path A. This necessitates that the microfluidic system adheres to specific geometric constraints, which will be elaborated on subsequently.

Consider the points X and Y upstream and downstream of the bypass respectively. Let ΔP_B be the pressure difference between X and Y passing through the loop (path B) of length L_B, and ΔP_A the pressure difference passing through the bypass (path A), whose restriction is of length L_A. These pressure differences do not depend on the chosen path, thus are equal.
In the Stokes regime, the pressure differences that occur are linear functions of the liquid flow. Microchannels have rectangular cross sections that induce regular pressure drops given by the equation[1]:

$$\Delta P = \frac{(w+h)^2}{2w^3h^3} P_0 \, \mu \, L \, Q \qquad (1)$$

Where Q is the flow-rate, μ is the dynamic viscosity of the liquid, w, L and h the width, length, and height respectively of the considered channel. P_O is a Poiseuille number characteristic of the cross-sectional geometry of the considered rectangular channel which can be expressed as a function of $\alpha = \frac{h}{w}$ the aspect ratio[2]:

$$P_O = 24(1 - 1.3553\,\alpha + 1.9467\,\alpha^2 - 1.7012\,\alpha^3 + 0.9564\,\alpha^4 - 0.2537\,\alpha^5) \qquad (2)$$

Consider the path B; the channel cross-section is square, hence $\alpha_B = 1$ and P_{O_B}=14.23, and the pressure difference is written (neglecting the additional pressure drop associated with the bend):

$$\Delta P_B = \frac{2\,P_{O_B}}{h^3}\,\mu\,Q_B\,\frac{L_B}{h} \qquad (3)$$

The pressure difference ΔP_A along path A can be considered as essentially resulting from the restriction. It is composed of a regular component, due to the friction of the flow along the lateral walls of the restriction of length L_A, and a singular component related to the change in cross-section of the flow at the entrance and exit of the restriction (contraction/expansion).

Concerning the regular component, by replacing α by α_A^{-1} in (2) for the expression of P_{O_A} (as gravity plays no role at these scales), one can write the expression for the regular pressure difference associated with path A:

$$\Delta P_{A_R} = \frac{\left(1+\frac{w_A}{h}\right)^2 P_{O_A}}{2w_A^3} \mu Q_A \frac{L_A}{h} \tag{4}$$

The singular pressure difference can be written as follows[3]:

$$\Delta P_{A_S} = \frac{(1-\gamma_A)K}{w_A^2 h} \mu Q_A \tag{5}$$

where we introduced $\gamma_A = \frac{w_A}{w_I}$ the contraction ratio of path A (w_I is the width of the entrance of the trap site, slightly larger than ϕ, the diameter of the organoid), and K, a coefficient that reflects the only effect of the aspect ratio:

$$K = \begin{cases} 9{,}70 + 18\left(\frac{w_A}{h}\right)^{1{,}1} & , \quad 0{,}33 < \frac{w_A}{h} < 0{,}8 \\ 10{,}15 & , \quad \frac{w_A}{h} < 0{,}33 \end{cases} \tag{6}$$

Since the pressure difference between points X and Y does not depend on the path followed, we have $\Delta P_B = \Delta P_A = \Delta P_{A_R} + \Delta P_{A_S}$ which translates to:

$$\frac{2P_{O_B}}{h^3} \mu Q_B \frac{L_B}{h} = \frac{\left(1+\frac{w_A}{h}\right)^2 P_{O_A}}{2w_A^3} \mu Q_A \frac{L_A}{h} + \frac{(1-\gamma_A)K}{w_A^2 h} \mu Q_A \tag{7}$$

Thus :

$$\frac{Q_B}{Q_A} = 0.035 \frac{h}{L_B}\left(\frac{h}{w_A}\right)^3 \left[\frac{1}{2}\left(1+\frac{w_A}{h}\right)^2 P_{O_A} \frac{L_A}{h} + K(1-\gamma_A)\frac{w_A}{h}\right] \tag{8}$$

Of note, this final expression does not contain any fluid velocity term, so the hydrodynamic trapping principle works for all velocities in the laminar regime. An organoid will be trapped if $Q_A > Q_B$, so the criterion for a functioning trap is $\frac{Q_B}{Q_A} < 1$.

Among these parameters, some are determined by the size of the spheroids considered; thus, h and w_I must be slightly larger than the diameter of the spheroid so that it can be introduced into the channel without deformation, switch into the converging part of the bypass, and then obstruct the restriction. In addition, L_A must be small enough to prevent an air bubble from getting trapped in the restriction due to capillarity during microchannel filling.

We still need to determine L_B and w_A for the trap to function. These are the two geometric parameters we can manipulate to dimension the microfluidic circuit. Supplementary Fig. 2d represents the function $\frac{Q_B}{Q_A} = f\left(\frac{L_B}{h}, \frac{w_A}{h}\right)$. Thus, the entire surface associated with $\frac{Q_B}{Q_A} < 1$ (dark blue and light blue) corresponds to a set of parameters compatible with the trapping system. On the contrary, the surface associated with $\frac{Q_B}{Q_A} > 1$ (orange and yellow) helps visualize situations that do not guarantee the trap's operation. For example, with a fixed L_B, the smaller w_A becomes, the closer the flow rate ratio approaches unity, as the pressure loss associated with the restriction increases. Similarly, with a fixed w_A, the smaller L_B becomes, the closer the flow rate ratio approaches unity, as the pressure loss associated with the loop decreases.

Finally, it's important to make sure not to drift too far from the $Q_A = Q_B$ line, in a way that the inequality $\frac{Q_B}{Q_A} < 1$ can easily be reversed to $\frac{Q_B}{Q_A} > 1$ after trapping the organoid. Indeed, in the microfluidic chip loading process described in this study, an air bubble is injected after the organoid is trapped. This bubble should preferably go through path B, otherwise, the encapsulation operation of the organoid can't be carried out. Similarly, note that drifting away from the $Q_A = Q_B$ line also poses a risk in a series setup of trapping units as defined in Supplementary Figure 4. Indeed, once an organoid is engaged in the bypass, the flow continues above the organoid, which does not block the entire height of the restriction. If, even after trapping the organoid, the inequality $\frac{Q_B}{Q_A} < 1$ persists, a second organoid could then be trapped in the same unit as the first one. It is therefore preferable to choose L_B and w_A in such a way that $\frac{Q_B}{Q_A}$ is slightly less than unity; this way, the inequality can be easily flipped after trapping an organoid, and the flow is then mainly redirected towards path B.

The dimensions that were practically chosen in this study are given below (Table 1):

	L_B/h	W_I/h	W_A/h	L_A/h	h (μm)	Q_B/Q_A
Mesenchymal and pancreatic islet spheroids (300 μm)	18.55	1.25	0.5	0.25	400	0.15
Blood vessel organoids (600 μm)	17.175	1.125	0.375	0.125	800	0.22

Table 1: Dimensions of the microfluidic chips used for vascular spheroids and organoids.

Supplementary Note 2: Reynolds number derivation (theory supplement)

The Reynolds number Re is given by: $Re = \frac{\rho v L}{\mu}$, where ρ is the density of the fluid, v is the flow speed, L is a characteristic length of the system and μ is the dynamic viscosity.

In our presented system, during the loading phase of the chip, we can choose $\rho \approx 1000$ kg/m^3, $L \approx 1$ mm, $v = \frac{Q}{S} \approx 5$ mm/s (using a flow rate of $Q = 300$ µl/min in a microchannel of cross-section S = 1 x 1 mm^2), and $\mu \approx 0.9$ mPa.s. This leads to a Reynolds number of $Re \approx 6$, ensuring a laminar regime.

During the long-term perfusion at $Q = 1$ µl/min, the fluid velocities in the vessels of radius $R = 15$ µm were measured at $v_{min} = 100$ µm/s and $v_{max} = 7500$ µm/s, thus corresponding to Reynolds number in the very low range of 10^{-3} to 10^{-1}.

Supplementary Note 3: Shear rate at the vessel wall derivation (theory supplement)

Assuming a steady laminar flow in a cylindrical blood vessel of radius R along a z-axis (where the radial and azimuthal components of the fluid velocity are zero, and the flow is axisymmetric; thus the pressure p is a function of the axial coordinate z only), the velocity profile is given by the Hagen-Poiseuille equation: $v(r) = \frac{R^2}{4\mu}\left(1 - \frac{r^2}{R^2}\right)\left|\frac{dp}{dz}\right|$ where μ is the dynamic viscosity of the fluid. The flow rate Q can be expressed as $Q = \int v(r)dA$ with $dA = 2\pi r dr$. Thus, $Q = \int_0^R \frac{R^2}{4\mu}\left|\frac{dp}{dz}\right|\left(1 - \frac{r^2}{R^2}\right) 2\pi r\, dr = \frac{\pi R^4}{8\mu}\left|\frac{dp}{dz}\right|$. Thus, the average velocity defined by $\bar{v} = \frac{Q}{\pi R^2}$ can be expressed as $\bar{v} = \frac{R^2}{8\mu}\left|\frac{dp}{dz}\right|$. Finally, one can find the shear rate at the blood vessel wall $\dot{\gamma_w} = \left.\frac{\partial v}{\partial r}\right|_{r=R} = \frac{4\bar{v}}{R}$.

Supplementary References

1. Bruus, H. *Theoretical microfluidics*. (Oxford University Press, 2007).
2. Shah, R. K. & London, A. L. Laminar Flow Forced Convection in Ducts. (1978).
3. Zivkovic, V., Zerna, P., Alwahabi, Z. T. & Biggs, M. J. A pressure drop correlation for low Reynolds number Newtonian flows through a rectangular orifice in a similarly shaped micro-channel. *Chem. Eng. Res. Des.* (2013) doi:10.1016/j.cherd.2012.05.022.
