# Supplementary material for: A microfluidic platform integrating functional vascularized organoids-on-chip
Source: Nat Commun. 2024 Feb 16;15:1452. doi: 10.1038/s41467-024-45710-4 (PMC10873332; doi:10.1038/s41467-024-45710-4)
Supplement: Supplementary file 3 — Description of additional supplementary files [file 41467_2024_45710_MOESM3_ESM.pdf]

DESCRIPTION OF ADDITIONAL SUPPLEMENTARY FILES

Supplementary Movie 1

Fluorescent microbeads perfusion in mesenchymal spheroid and neighboring endothelial network. Raw images (greyscale). The second movie displays only the objects in motion, which is achieved through successive image subtraction processes.

Supplementary Movie 2

Processed images of fluorescent microbeads perfusion in mesenchymal spheroid and neighboring endothelial network after using the skeletonize feature in ImageJ. The second and third movies present the evolution of pixels using a color code that corresponds to time. The third movie shows a close-up view at the entrance of the trap.

Supplementary Movie 3

Fluorescent microbeads perfusion in mesenchymal spheroid (higher magnification). Raw images (green and red fluorescence). The second movie shows a close-up view of a HUVEC vessel perfused by 0.5 µm and 4.8 µm microbeads. The third movie shows the perfusion of the mesenchymal endothelium (green) by one 3.2 µm microbead (red).

Supplementary Movie 4

Individual microbead passing through the GFP-HUVEC network arborizing blood vessel organoid. Raw images (green and red fluorescence). The second movie shows several 3.2 µm microbeads (red) passing through the endothelial network (green) and choosing different paths within the network.

Supplementary Movie 5

Individual microbeads flowing through the blood vessel organoid's vasculature. Raw images (blue, green and red fluorescence). The two movies show microbeads (cyan) flowing within the blood vessel organoid's vasculature at two different areas and z-planes. The observed pulsatile movement of the tissue is attributed to the strong pulses imposed by the syringe pump used to flow the beads, which was done to enhance the chances of observing beads perfuse the narrower vessels of the BVOs within a limited timeframe, minimizing photobleaching.
