# Supplementary material for: A microfluidic platform integrating functional vascularized organoids-on-chip
Source: Nat Commun. 2024 Feb 16;15:1452. doi: 10.1038/s41467-024-45710-4 (PMC10873332; doi:10.1038/s41467-024-45710-4)
Supplement: Supplementary file 9 — Reporting Summary [file 41467_2024_45710_MOESM9_ESM.pdf]

nature portfolio

Corresponding author(s): Josef M. Penninger, Xavier Gidrol

Last updated by author(s): Jan 9, 2024

Reporting Summary

Nature Portfolio wishes to improve the reproducibility of the work that we publish. This form provides structure for consistency and transparency in reporting. For further information on Nature Portfolio policies, see our Editorial Policies and the Editorial Policy Checklist.

Statistics

For all statistical analyses, confirm that the following items are present in the figure legend, table legend, main text, or Methods section.

n/a	Confirmed	
☐	☒	The exact sample size (*n*) for each experimental group/condition, given as a discrete number and unit of measurement
☐	☒	A statement on whether measurements were taken from distinct samples or whether the same sample was measured repeatedly
☐	☒	The statistical test(s) used AND whether they are one- or two-sided<br>*Only common tests should be described solely by name; describe more complex techniques in the Methods section.*
☒	☐	A description of all covariates tested
☐	☒	A description of any assumptions or corrections, such as tests of normality and adjustment for multiple comparisons
☐	☒	A full description of the statistical parameters including central tendency (e.g. means) or other basic estimates (e.g. regression coefficient) AND variation (e.g. standard deviation) or associated estimates of uncertainty (e.g. confidence intervals)
☐	☒	For null hypothesis testing, the test statistic (e.g. *F*, *t*, *r*) with confidence intervals, effect sizes, degrees of freedom and *P* value noted<br>*Give P values as exact values whenever suitable.*
☒	☐	For Bayesian analysis, information on the choice of priors and Markov chain Monte Carlo settings
☒	☐	For hierarchical and complex designs, identification of the appropriate level for tests and full reporting of outcomes
☒	☐	Estimates of effect sizes (e.g. Cohen's *d*, Pearson's *r*), indicating how they were calculated

Our web collection on statistics for biologists contains articles on many of the points above.

Software and code

Policy information about availability of computer code

Data collection	Samples were imaged using standard brightfield and fluorescence microscopy (Olympus IX50), or confocal microscopy for immunostained samples (Nikon Eclipse Ti-E Spinning Disk microscope, Zeiss LSM880 scanning confocal microscope, and Leica SP8 scanning confocal microscope). For microbeads perfusion in the BVOs' vasculature, since we needed to capture images deep within the tissue at a high frame rate while minimizing photobleaching, confocal microscopy was unsuitable. Therefore, we employed a Leica THUNDER 3D Cell Imager microscope for this purpose. For bulk RNA sequencing, RNA extraction was done using the Trizol protocol (Invitrogen). Sample quality control was performed using the Agilent 2100 Bioanalyzer to ensure high-quality RNA input. Qualifying samples underwent library preparation following the standard protocol for the Illumina Stranded mRNA prep (Illumina). Subsequently, sequencing was performed on the Illumina NextSeq2000 platform.
Data analysis	Data analysis was performed using standard commands in Excel 2019 and plotting/statistics were performed in GraphPad Prism 9 software. The Fiji (ImageJ) plugin Angiogenesis Analyzer was used for networks quantifications. Imaris 9 software was used for 3D analysis of the confocal z-stacks. Raw sequence reads of RNA sequencing and data plotting were processed using RStudio 4.3.2, R/Bioconductor package AnnotationDbi (v.1.60.0), R/Bioconductor package DESeq2 (v.1.3.8.3), R/Bioconductor package ComplexHeatmap (v.2.14.0), R/Bioconductor package clusterProfiler (v.4.6.0), R/Bioconductor package RUVSeq (v.1.32.0). All the codes used for this study are available at: https://github.com/ClementQuintard/A-microfluidic-platform-integrating-functional-vascularized-organoids-on-chip.

For manuscripts utilizing custom algorithms or software that are central to the research but not yet described in published literature, software must be made available to editors and reviewers. We strongly encourage code deposition in a community repository (e.g. GitHub). See the Nature Portfolio guidelines for submitting code & software for further information.

Data

Policy information about availability of data

All manuscripts must include a data availability statement. This statement should provide the following information, where applicable:

- Accession codes, unique identifiers, or web links for publicly available datasets
- A description of any restrictions on data availability
- For clinical datasets or third party data, please ensure that the statement adheres to our policy

All the numeric data in this study are included in the Source Data files. The raw images are too large for public deposit and are available from the corresponding authors on reasonable request. RNA-seq data has been deposited to NCBI and is accessible with the following: https://www.ncbi.nlm.nih.gov/sra/PRJNA1061525. All the codes used for this study are available at: https://github.com/ClementQuintard/A-microfluidic-platform-integrating-functional-vascularized-organoids-on-chip.

Research involving human participants, their data, or biological material

Policy information about studies with human participants or human data. See also policy information about sex, gender (identity/presentation), and sexual orientation and race, ethnicity and racism.

Reporting on sex and gender	*Use the terms sex (biological attribute) and gender (shaped by social and cultural circumstances) carefully in order to avoid confusing both terms. Indicate if findings apply to only one sex or gender; describe whether sex and gender were considered in study design; whether sex and/or gender was determined based on self-reporting or assigned and methods used. Provide in the source data disaggregated sex and gender data, where this information has been collected, and if consent has been obtained for sharing of individual-level data; provide overall numbers in this Reporting Summary. Please state if this information has not been collected. Report sex- and gender-based analyses where performed, justify reasons for lack of sex- and gender-based analysis.*
Reporting on race, ethnicity, or other socially relevant groupings	*Please specify the socially constructed or socially relevant categorization variable(s) used in your manuscript and explain why they were used. Please note that such variables should not be used as proxies for other socially constructed/relevant variables (for example, race or ethnicity should not be used as a proxy for socioeconomic status). Provide clear definitions of the relevant terms used, how they were provided (by the participants/respondents, the researchers, or third parties), and the method(s) used to classify people into the different categories (e.g. self-report, census or administrative data, social media data, etc.) Please provide details about how you controlled for confounding variables in your analyses.*
Population characteristics	*Describe the covariate-relevant population characteristics of the human research participants (e.g. age, genotypic information, past and current diagnosis and treatment categories). If you filled out the behavioural & social sciences study design questions and have nothing to add here, write "See above."*
Recruitment	*Describe how participants were recruited. Outline any potential self-selection bias or other biases that may be present and how these are likely to impact results.*
Ethics oversight	*Identify the organization(s) that approved the study protocol.*

Note that full information on the approval of the study protocol must also be provided in the manuscript.

Field-specific reporting

Please select the one below that is the best fit for your research. If you are not sure, read the appropriate sections before making your selection.

☒ Life sciences ☐ Behavioural & social sciences ☐ Ecological, evolutionary & environmental sciences

For a reference copy of the document with all sections, see nature.com/documents/nr-reporting-summary-flat.pdf

Life sciences study design

All studies must disclose on these points even when the disclosure is negative.

Sample size	Sample size for every experiment is reported in the figure legends or in the Methods section under "Statistics and Reproducibility". For quantitative analyzes, sample size was determined based on similar studies in the field, and no sample size calculation was performed. For each experiment, a minimum of 3 biological replicates were used as it is standard in the field. For every experiment, the sample size was sufficient four our claims based on statistical significance.
Data exclusions	None.
Replication	Each experiment described in this study was repeated at least 3 times. We confirmed that the experimental findings were reliably reproduced between experiments.
Randomization	Organoids were randomly allocated into the different experimental groups (wells, static, flow).

Blinding	Blinding was not possible as the same investigator processed the organoids and analyzed the data. However, we used in this study well-defined standardized methods and quantitative metrics which do not involve any subjective measurements.

Reporting for specific materials, systems and methods

We require information from authors about some types of materials, experimental systems and methods used in many studies. Here, indicate whether each material, system or method listed is relevant to your study. If you are not sure if a list item applies to your research, read the appropriate section before selecting a response.

Materials & experimental systems

n/a	Involved in the study
☐	☒ Antibodies
☐	☒ Eukaryotic cell lines
☒	☐ Palaeontology and archaeology
☒	☐ Animals and other organisms
☒	☐ Clinical data
☒	☐ Dual use research of concern
☒	☐ Plants

Methods

n/a	Involved in the study
☒	☐ ChIP-seq
☒	☐ Flow cytometry
☒	☐ MRI-based neuroimaging

Antibodies

Antibodies used

Primary antibodies
Antibody, Catalog number, Host species, Concentration
CD31, Abcam, ab134168, rabbit anti-human, 1:200
CD31, Abcam, ab9498, mouse anti-human, 1:200
PDGFRβ, Cell Signaling Technology, 3169S, rabbit anti-human, 1:200
ColIV, Chemicon, AB769, goat anti-human, 1:50
SM22/TAGLN, Abcam, ab14106, rabbit anti-human, 1:200
ZO-1, Abcam, ab216880, rabbit anti-human, 1:200
VE-Cadherin, Abcam, ab33168, rabbit anti-human, 1:200

Secondary antibodies
donkey anti-mouse Alexa Fluor 488, Invitrogen, A-21202
donkey anti-rabbit Cy3 Jackson ImmunoResearch Inc., 711-165-152
donkey anti-rabbit Alexa Fluor 555, Invitrogen, A-31572
donkey anti-rabbit Alexa Fluor 647, Invitrogen, A-31573
donkey anti-goat Alexa Fluor 555, Invitrogen, A-21432
donkey anti-goat and Alexa Fluor 647, Invitrogen, A-21447

Validation

All antibodies used were validated for immunofluorescence staining according to manufacturers' websites. Validation statements for the species and application on the manufacturers' websites are listed below. Antibodies were further chosen based on having references in the literature, and include many antibodies used and reported in our labs prior work.
ab134168: Rabbit monoclonal [EP3095] to CD31, Suitable for: Flow Cyt (Intra), IHC-P, WB, Reacts with: Human
ab9498: Mouse monoclonal [JC/70A] to CD31, Suitable for: IHC-Fr, WB, IHC-P, Flow Cyt, ICC/IF, Reacts with: Human
3169S: Rabbit monoclonal to PDGFRβ, Suitable for: WB, IP, IHC, ChIP, C&R-, C&T, DB, eCLIP, IF, F, Reacts with: Human, Mouse, Rat
AB769: Goat polyclonal to ColIV, Suitable for: ELISA, DB, IHC, Reacts with: Human, Bovine, Mouse
ab14106: Rabbit polyclonal to TAGLN/Transgelin, Suitable for: ICC/IF, WB, Reacts with: Mouse, Rat, Human

Eukaryotic cell lines

Policy information about cell lines and Sex and Gender in Research

Cell line source(s)	The iPSC line NC8 was provided by Manfred Boehm, NIH.<br>The primary human fibroblasts (FMA73) line was provided by Walid Rachidi, CEA Grenoble.<br>The HUVEC lines were purchased from Amgio-Proteomie.
Authentication	None of the cell lines were authenticated.
Mycoplasma contamination	All cell lines used in this study were tested negative for mycoplasma contamination on a monthly routine.
Commonly misidentified lines (See ICLAC register)	No commonly misidentified cell lines were used in this study.

Plants

Seed stocks	*Report on the source of all seed stocks or other plant material used. If applicable, state the seed stock centre and catalogue number. If plant specimens were collected from the field, describe the collection location, date and sampling procedures.*
Novel plant genotypes	*Describe the methods by which all novel plant genotypes were produced. This includes those generated by transgenic approaches, gene editing, chemical/radiation-based mutagenesis and hybridization. For transgenic lines, describe the transformation method, the number of independent lines analyzed and the generation upon which experiments were performed. For gene-edited lines, describe the editor used, the endogenous sequence targeted for editing, the targeting guide RNA sequence (if applicable) and how the editor was applied.*
Authentication	*Describe any authentication procedures for each seed stock used or novel genotype generated. Describe any experiments used to assess the effect of a mutation and, where applicable, how potential secondary effects (e.g. second site T-DNA insertions, mosiacism, off-target gene editing) were examined.*
